# Constrained Multiple Planar Reconstruction for Automatic Camera Calibration of Intelligent Vehicles

**DOI:** 10.3390/s21144643

**Published:** 2021-07-06

**Authors:** Sang Jun Lee, Jae-Woo Lee, Wonju Lee, Cheolhun Jang

**Affiliations:** 1 Division of Electronic Engineering, Jeonbuk National University, 567 Baekje-daero, Deokjin-gu, Jeonju-si 54896, Jeollabuk-do, Korea; sj.lee@jbnu.ac.kr; 2 Samsung Advanced Institute of Technology (SAIT), 130 Samsung-ro, Yeongtong-gu, Suwon-si 16678, Gyeonggi-do, Korea; wonjulee@kaist.ac.kr (W.L.); c_h.jang@samsung.com (C.J.)

**Keywords:** computer vision, intelligent vehicles, extrinsic camera calibration, structure from motion, convex optimization

## Abstract

In intelligent vehicles, extrinsic camera calibration is preferable to be conducted on a regular basis to deal with unpredictable mechanical changes or variations on weight load distribution. Specifically, high-precision extrinsic parameters between the camera coordinate and the world coordinate are essential to implement high-level functions in intelligent vehicles such as distance estimation and lane departure warning. However, conventional calibration methods, which solve a Perspective-n-Point problem, require laborious work to measure the positions of 3D points in the world coordinate. To reduce this inconvenience, this paper proposes an automatic camera calibration method based on 3D reconstruction. The main contribution of this paper is a novel reconstruction method to recover 3D points on planes perpendicular to the ground. The proposed method jointly optimizes reprojection errors of image features projected from multiple planar surfaces, and finally, it significantly reduces errors in camera extrinsic parameters. Experiments were conducted in synthetic simulation and real calibration environments to demonstrate the effectiveness of the proposed method.

## 1. Introduction

Recovering the positions of 3D points from 2D-2D correspondences is a fundamental building block in geometric computer vision. This is called triangulation, and it is an essential procedure for many applications including structure-from-motion (SfM) [1,2,3], simultaneous localization and mapping (SLAM) [4,5,6], and visual odometry [7,8]. Triangulation is conducted based on displacements between image correspondences obtained from stereo cameras or a moving camera. In an ideal case, back-projected rays from an image correspondence intersect at a point in three dimensional space, and it can be simply formulated by a direct linear transformation. However, in practice, the rays do not necessarily intersect due to measurement noise involved in image features, and these features do not in general satisfy the epipolar geometry [9]. Therefore, recovering 3D information is not a trivial problem even in a two-view case.

A standard approach for addressing the problem of noisy measurements is to estimate 2D corrected correspondences which satisfy the epipolar geometry with the minimum geometric cost [9,10,11]. These 2D corrected points are maximum likelihood estimates under the assumption of zero-mean isotropic Gaussian noise on the measurements [10]. Triangulation is conducted for the corrected correspondences, and it is equivalent to estimate 3D points which minimize the reprojection error. This procedure is called optimal triangulation. In case that all the 3D points are on a plane, their projected points in two views are associated with a projective transformation which is called homography [12]. Chum et al. proposed a method to find optimal 2D correspondences projected from 3D points on a plane, and it is called optimal planar reconstruction [13]. Kanatani et al. further derived an optimal solution for planar scene triangulation in case that plane and camera parameters are unknown [14]. Planar constraint in general reduces a significant amount of reconstruction error by associating multiple image features for correcting individual 2D measurements. This motivates us to associate image features projected from multiple planar surfaces to improve the precision of 3D reconstruction.

In this paper, we propose a multiple planar reconstruction method which can be applicable in a man-made environment: planes of interest are perpendicular to the ground. It is worth noting that this scenario is quite reasonable in environments for end-of-line calibration and indoor camera calibration. This assumption implies that the normal vectors of planes are coplanar. Whereas previous methods reconstruct individual planes, we seek to jointly optimize the structures of multiple planes simultaneously by introducing coplanarity constraints of their normal vectors.

Another main work of this paper is a novel extrinsic camera calibration method. Among various types of extrinsic parameters, our goal is to estimate extrinsic parameters between the camera coordinate and the world coordinate, which are essential prerequisite for high-level functions in intelligent vehicles such as distance estimation and lane departure warning. Extrinsic parameters consist of relative rotation and translation between the camera and world coordinates, and the world coordinate is also called the vehicle coordinate. In recent intelligent vehicles, due to unpredictable mechanical changes or variations on weight distribution, it is desirable to perform extrinsic camera calibration on a regular basis [15], e.g., at the start of every driving. However, camera calibration requires correspondences between 2D image projections and their 3D points [16] to solve a variant of Perspective-n-Point (PnP) problems [17,18,19,20,21], and the procedure for measuring accurate 3D points in the world coordinate is the biggest bottleneck in conventional methods.

Recently, deep learning methods have been utilized in automatic camera calibration for intelligent vehicles [22,23,24]. However, many original equipment manufacturers (OEMs) and Tier 1 component companies require conventional computer vision methods to guarantee the safety and reliability of the camera calibration function. Moreover, even OEMs, which employ deep learning techniques in recognition and planning algorithms, parallelly utilize conventional methods to acquire a satisfactory level of functional safety for several essential functions such as camera calibration. This paper proposes an extrinsic camera calibration method to reduce manual work in conventional approaches. The proposed method uses 3D cues in the camera coordinate to infer 3D information in the world coordinate by utilizing 3D points on a planar chessboard which is perpendicular to the ground. The main advantage of the method is that it is able to estimate extrinsic parameters without measuring 3D points in the world coordinate.

The contributions of this paper are twofold: (1) multiple planar reconstruction method to jointly recover 3D structures of multiple planar surfaces and (2) extrinsic camera calibration method based on the reconstructed points. This paper is organized as follows. Section 2 presents the multiple planar reconstruction method, and Section 3 explains the extrinsic camera calibration method based on 3D reconstruction. Section 4 presents experimental results in both synthetic and real environments to demonstrate the effectiveness of the proposed methods.

## 2. Optimal Multiple-Planar Reconstruction

**Figure 1:**
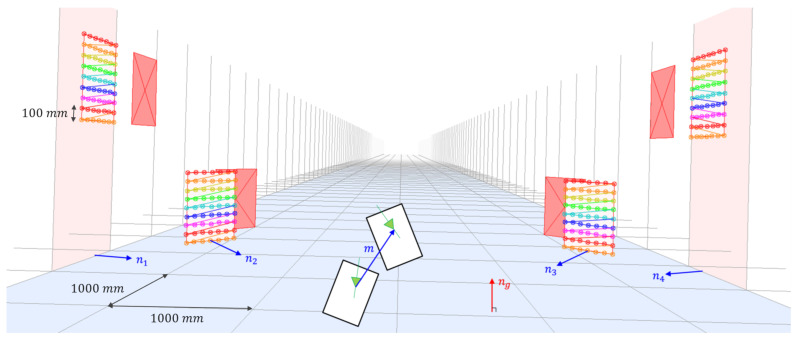
Synthetic simulation environment for multiple planar reconstruction and camera extrinsic calibration.

This section presents the proposed method for joint reconstruction of multiple planar surfaces. We assume that planar chessboards are installed perpendicular to the ground, and a vehicle with a camera moves along the ground with a planar motion. Under these assumptions, the plane normal vectors and camera motion vector are orthogonal to the ground normal vector, and therefore, the plane normal and camera motion vectors are coplanar. Although these assumptions are not easy to satisfy in a road situation, it is worth utilizing the proposed method to improve the precision and robustness of end-of-line and indoor calibrations, which are usually conducted in a man-made environment. In Figure 1, a synthetic configuration containing four planar boards in three dimensional space is projected onto an image plane by using a camera matrix and arbitrary extrinsic parameters. The camera motion vector is indicated by m, and the tetragons filled with a reddish color and the tetragons filled with 2D points depict the projections of the four planar boards from the first and second views, respectively.

The outline of the proposed method is as follows. First, normal vectors of the planar chessboards are jointly optimized to minimize the algebraic error of homographies subject to the coplanarity constraints. Then, these homographies are used to correct 2D measurements, and 3D structures of the planar surfaces are recovered via triangulation of the corrected 2D correspondences. Finally, extrinsic parameters are estimated by using the method presented in Section 3.

### 2.1. Plane Induced Homographies

Suppose that planar surfaces are imaged by a calibrated camera in two views under planar motion of a vehicle. In three dimensional space, the *k*-th plane can be represented as its scaled normal vector $n_{k}$ so that $n_{k}^{⊤}X_{C}+1=0$, where $X_{C}$ is a 3D point in the camera coordinate. Suppose that the essential matrix between the two-view is given by $E={\left[t\right]}_{\times }R$, where R is rotation matrix, t is translation vector, and ${\left[\cdot \right]}_{\times }$ is a 3×3 skew-symmetric matrix for representing cross product as a matrix multiplication. Then, it is well-known that a homography induced by the *k*-th plane can be represented as

(1) $$H_{k}=R-tn_{k}^{⊤}.$$

The essential matrix, R, and t can be computed via ego-motion estimation. There are several methods to estimate ego-motion of a vehicle, and many previous methods utilize optical flow of background features [25,26].

Suppose that the *i*-th point on the *k*-th plane in three dimensional space is projected onto two image planes, and $x_{i,k}$ and $x_{i,k}^{\prime }$ are homogeneous representations of the 2D projections in the first and second views, respectively. Then the 2D correspondence satisfies $x_{i,k}^{\prime }≃H_{k}x_{i,k}=\left(R-tn_{k}^{⊤}\right)x_{i,k}$, and it can be reformulated as

(2) $$x_{i,k}^{⊤}n_{k}=\frac{{\left(x_{i,k}^{\prime }\times Rx_{i,k}\right)}^{⊤}\left(x_{i,k}^{\prime }\times t\right)}{{\left(x_{i,k}^{\prime }\times t\right)}^{⊤}\left(x_{i,k}^{\prime }\times t\right)}=b_{i,k},$$

when $x_{i,k}^{\prime }$ and t are not parallel. The operation × indicates cross-product between two vectors, and ≃ implies that two vectors are equal up to scale. The scaled normal vector $n_{k}$ of the *k*-th plane can be obtained by solving the unconstrained optimization problem:

(3) $$\begin{array}{cccc} \; & \underset{n_{k}}{\mathrm{minimize}}\; & \; & ∥A_{k}n_{k}-b_{k}{∥}^{2}, \end{array}$$

where $b_{k}={\left[b_{1,k},\cdots ,b_{N_{k},k}\right]}^{⊤}\in R^{N_{k}}$ and $A_{k}={\left[x_{1,k},\cdots ,x_{N_{k},k}\right]}^{⊤}\in R^{N_{k}\times 3}$. Homography optimization in the previous work [14] can be reformulated as a two-step process: optimization of a scaled normal vector by (3) and homography recovery by (1).

### 2.2. Multiple Planar Reconstruction

This section presents the main idea of the proposed method which introduces coplanarity constraints of plane normal vectors and camera motion vector to jointly reconstruct multiple planar surfaces. In three dimensional space, coplanarity of the normal vectors $n_{i}$ (i=1,⋯,K) and camera motion $m=-R^{⊤}t$ can be represented as

(4) $$(n_{1}\times n_{k})\cdot m=0,\;\;\;k=2,\cdots ,K.$$

To formulate the quadratic constraints in terms of optimization variables, all of the constraints are associated with the camera motion vector m, which is constant in the optimization problem. The number of equations for constraining the coplanarity of *K* normal vectors and m is K(K+1)/2. However, these equations have redundancy, because, for example, the coplanarities of $\left(n_{i},n_{j},m\right)$ and $\left(n_{i},n_{k},m\right)$ ensure the coplanarity of $\left(n_{j},n_{k},m\right)$. Therefore, the minimum number of equality constraints for the coplanarity of *K* normal vectors is K−1.

Let w be a concatenated normal vector such that $w={\left[n_{1}^{⊤},\cdots ,n_{K}^{⊤}\right]}^{⊤}$, then the coplanarity (4) can be reformulated as

(5) $$w^{⊤}C_{k}w=0,\;\;\;k=2,\cdots ,K,$$

where $C_{k}$ is a 3K×3K symmetric block matrix, of which partitions are 3×3 zero matrices except that $C_{1k}={\left[m\right]}_{\times }$ and $C_{k1}={\left[m\right]}_{\times }^{⊤}$; $C_{ij}$ is a 3×3 submatrix corresponding to the *i*-th row and *j*-th column block. By introducing an auxiliary dimension to w so that $\tilde{w}={\left[w^{⊤},1\right]}^{⊤}$, the optimization problem for minimizing the objective function of (3) subject to the coplanarity constraints (4) can be formulated as

(6) $$\begin{array}{cccc} \; & \underset{\tilde{w}}{\mathrm{minimize}}\; & \; & {\tilde{w}}^{⊤}Q\tilde{w}\\ \; & \mathrm{subject}\;\mathrm{to}\; & \; & {\tilde{w}}^{⊤}{\tilde{C}}_{k}\tilde{w}=0,\;\;\;k=2,\cdots ,K,\\ \; & \; & & {\tilde{w}}^{⊤}C_{0}\tilde{w}=1, \end{array}$$

where $Q=\left[\begin{array}{cc} {\tilde{A}}^{⊤}\tilde{A} & -{\tilde{A}}^{⊤}b\\ -b^{⊤}\tilde{A} & 0 \end{array}\right]$, ${\tilde{C}}_{k}=\left[\begin{array}{cc} C_{k} & 0\\ 0^{⊤} & 0 \end{array}\right]$, $C_{0}=\left[\begin{array}{cc} 0_{3K} & 0\\ 0^{⊤} & 1 \end{array}\right]$, $\tilde{A}\in R^{(\sum_{k=1}^{K}N_{k})\times 3}$ is the block diagonal matrix, of which the *k*-th diagonal submatrix is $A_{k}\in R^{N_{k}\times 3}$ and off-diagonal blocks are zero matrices, and $0_{3K}$ is a 3K×3K zero matrix. This optimization problem (6) is a quadratically constrained quadratic program (QCQP); the objective is a quadratic function with a positive semidefinite matrix, and the constraints are quadratic with symmetric matrices. Becuase (6) is an NP-hard optimization problem, we reformulate it as a semidefinite program (SDP) by applying the parameterization of $S=\tilde{w}{\tilde{w}}^{⊤}$ and relaxation of a rank constraint.

### 2.3. SDP Relaxation

With the parametrization of $S=\tilde{w}{\tilde{w}}^{⊤}\in S_{+}$, where $S_{+}$ is the set of positive semidefinite matrices, the QCQP (6) can be reformulated in terms of inner products of matrices as

(7) $$\begin{array}{cccc} \; & \underset{S\in S_{+}}{\mathrm{minimize}}\; & \; & \langle Q\;,S\rangle \\ \; & \mathrm{subject}\;\mathrm{to}\; & \; & \langle {\tilde{C}}_{k}\;,S\rangle =0,\;\;\;k=2,\cdots ,K,\\ \; & \; & & \langle C_{0}\;,S\rangle =1,\\ \; & \; & & rank(S)=1. \end{array}$$

By eliminating the rank constraint in (7), we can obtain the semidefinite relaxation:

(8) $$\begin{array}{cccc} \; & \underset{S\in S_{+}}{\mathrm{minimize}}\; & \; & \langle Q\;,S\rangle \\ \; & \mathrm{subject}\;\mathrm{to}\; & \; & \langle \tilde{C}\;,S\rangle =0,\;\;\;k=2,\cdots ,K,\\ \; & \; & & \langle C_{0}\;,S\rangle =1. \end{array}$$

Becuase the SDP (8) is a convex optimization, we can find the global optimum reliably. Zhao proved the tightness between a primal QCQP and its rank relaxation at noise-free observations, and further showed the stability of rank relaxation at noisy observations [27]. We indeed observe that the solution of rank-relaxed problem (8) always satisfies the rank-1 in both synthetic simulation and real calibration environments.

### 2.4. Recovering 3D Points

**Figure 2:**
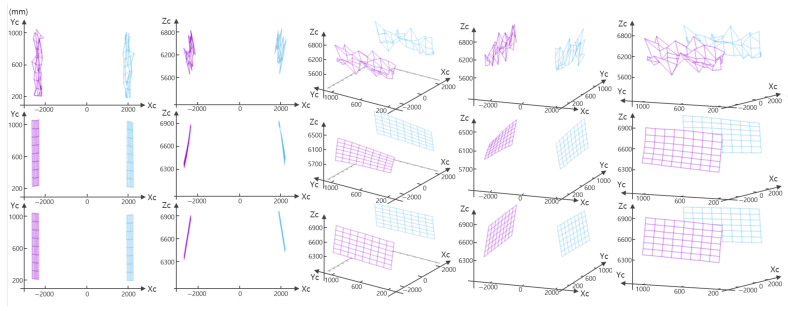
Reconstructed 3D points by using Naïve triangulation (**top** row), optimal planar reconstruction (**middle** row), and proposed reconstruction method (**bottom** row) in different viewpoints.

Once the optimal S of the SDP (8) is obtained, $\tilde{w}$ can be recovered by computing the eigenvector of S corresponding to the largest eigenvalue. By utilizing $\tilde{w}$ and ego-motion of the vehicle, individual homographies are computed by (1). Based on the plane induced homographies, optimal corrections ${\hat{x}}_{i,k}$ and ${\hat{x}}_{i,k}^{\prime }$ can be obtained for each 2D measurements $x_{i,k}$ and $x_{i,k}^{\prime }$, by solving a polynomial of degree 8 [13] or by using Sampson’s method [28]. The positions of 3D points can be recovered by applying triangulation to the corrected 2D points ${\hat{x}}_{i,k}$ and ${\hat{x}}_{i,k}^{\prime }$. Figure 2 shows 3D points on planar surfaces reconstructed by naïve triangulation [28], optimal planar reconstruction [13], and our method.

## 3. Camera Calibration Based on 3D Reconstruction

### 3.1. Vehicle Modeling

**Figure 3:**
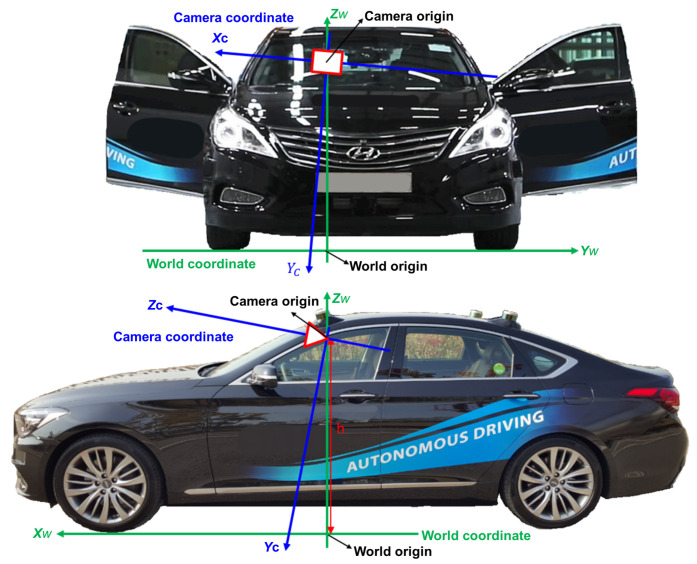
The definition of camera and world coordinates in our vehicle model.

In this section, we propose a monocular camera calibration method based on 3D reconstruction. Figure 3 shows our vehicle model. Our world coordinate is defined so that its origin is the perpendicular projection of the camera centre to the ground, and the direction of $Z_{W}$ axis is identical to the normal vector of the world coordinate so that it follows ISO 8855. Under the definition of the world coordinate, fixed values of longitudinal and lateral offsets between the world origin and the foremost point of a vehicle can be compensated at the process of generating signals such as distances to frontal vehicles and time to collision.

The relation between the world and camera coordinates can be formulized in terms of Euler angles (pitch θ, yaw ψ, roll ϕ) and camera height (*h*) as

(9) $$X_{C}=R\left(\theta ,\phi ;\psi \right)X_{W}+t\left(\theta ,\phi ,h;\psi \right),$$

where $X_{C}={\left[X_{C},Y_{C},Z_{C}\right]}^{⊤}$ is a 3D point in the camera coordinate, $X_{W}={\left[X_{W},Y_{W},Z_{W}\right]}^{⊤}$ is a 3D point in the world coordinate, and the rotation matrix R(θ,ϕ;ψ) is defined as (10).

(10) $$R\left(\theta ,\phi ;\psi \right)=\left[\begin{array}{ccc} \cos\theta \sin\psi \cos\phi +\sin\theta \sin\phi & -\cos\psi \cos\phi & -\sin\theta \sin\psi \cos\phi +\cos\theta \sin\phi \\ \cos\theta \sin\psi \sin\phi -\sin\theta \cos\phi & -\cos\psi \sin\phi & -\sin\theta \sin\psi \sin\phi -\cos\theta \cos\phi \\ \cos\theta \cos\psi & \sin\psi & -\sin\theta \cos\psi \end{array}\right].$$

Since $0=R\left(\theta ,\phi ;\psi \right)\cdot {\left[0,0,h\right]}^{⊤}+t\left(\theta ,\phi ,h;\psi \right)$, the translation can be represented as

(11) $$t\left(\theta ,\phi ,h;\psi \right)=-r_{3}h,$$

where $r_{i}$ is the *i*-th column vector of R(θ,ϕ;ψ).

Suppose that 3D world points of interest are on rectangular planar boards, which are perpendicular to the ground, and their $Z_{W}$ components (height) are measured beforehand in the world coordinate. Image features projected from these 3D points are detected while a vehicle with a camera moves along the ground, and yaw angle of the camera is estimated by computing a focus of expansion as presented in [29]. The 3D positions corresponding to these image features are recovered in the camera coordinate by using the multiple planar reconstruction method which is explained in Section 2. The objective of the automatic calibration algorithm is to estimate pitch (θ), roll (ϕ), and camera height (*h*) to recover relative rotation and translation between the camera and world coordinates.

### 3.2. Estimation of Extrinsic Parameters

Let $X_{W}^{i}$ and $X_{W}^{j}$ be the 3D points on a vertical line which is perpendicular to the ground. Since $X_{W}^{i}-X_{W}^{j}=0$ and $Y_{W}^{i}-Y_{W}^{j}=0$, component-wise differences between $X_{W}^{i}$ and $X_{W}^{j}$ can be simplified as (12).

(12) $$\begin{array}{cc} X_{C}^{i}-X_{C}^{j} & =-\left(\sin\theta \sin\psi \cos\phi -\cos\theta \sin\phi \right)\left(Z_{W}^{i}-Z_{W}^{j}\right),\\ Y_{C}^{i}-Y_{C}^{j} & =-\left(\sin\theta \sin\psi \sin\phi +\cos\theta \cos\phi \right)\left(Z_{W}^{i}-Z_{W}^{j}\right),\\ Z_{C}^{i}-Z_{C}^{j} & =-\sin\theta \cos\psi (Z_{W}^{i}-Z_{W}^{j}). \end{array}$$

Based on (12), pitch angle (θ) can be estimated by

(13) $$\sin\theta =-\frac{Z_{C}^{i}-Z_{C}^{j}}{\cos\psi (Z_{W}^{i}-Z_{W}^{j})}.$$

By solving $X_{C}^{i}$ and $Y_{C}^{i}$ in terms of sinϕ, roll angle (ϕ) can be estimated by

(14) $$\sin\phi =\frac{\cos\theta \left(X_{C}^{i}-X_{C}^{j}\right)-\sin\theta \sin\psi \left(Y_{C}^{i}-Y_{C}^{j}\right)}{\left(\sin^{2}\theta \sin^{2}\psi +\cos^{2}\theta \right)\left(Z_{W}^{i}-Z_{W}^{j}\right)}.$$

After the computation of θ and ϕ, camera height (*h*) can be obtained by solving the following equation with respect to $X_{W}$, $Y_{W}$, and *h*:

(15) $$\left[r_{1},r_{2},-r_{3}\right]{\left[X_{W},Y_{W},h\right]}^{⊤}=X_{C}-r_{3}Z_{W}.$$

Finally, camera extrinsic parameters can be recovered by using (10) and (11).

## 4. Experimental Results

The proposed method is composed of constrained multiple planar reconstruction and automatic extrinsic camera calibration. To demonstrate the effectiveness of each method, we synthesized a simulation environment, and the reconstruction and calibration errors were evaluated step by step. In both simulation and real experiments, Naïve triangulation [28] and optimal planar reconstruction method [13] were compared with the proposed method. To analyze the effect of the coplanarity constraint, we evaluate the proposed method with two experimental setups: the coplanarity of two plane normal vectors (K=2) and the coplanarity of four plane normal vectors (K=4). For fairness, we used all of the 3D points on the four planar surfaces in every reconstruction method. For example, in the case of K=2, two SDPs were optimized to use all of the image features projected from the four planar surfaces. The reconstruction and camera height errors were measured in millimetre (mm), and rotation errors were measured in degree.

### 4.1. Synthetic Environment

**Figure 4:**
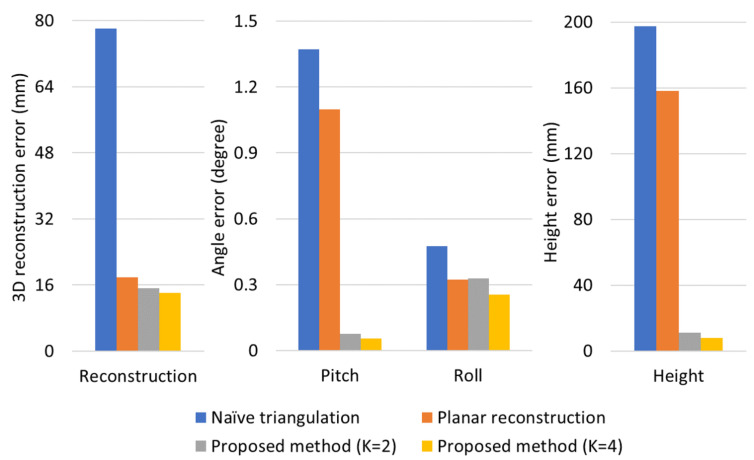
Reconstruction and calibration errors in simulation experiments.

To generate a simulation environment, camera extrinsic parameters were randomly sampled under the normal distributions: θ, ψ, ϕ∼$N(0,\;1^{2})$ and *h*∼$N(1300,\;50^{2})$, where N is normal distribution with a given mean and variance. This synthetic environment reflects the variations of real extrinsic parameters in our vehicle model, and degree and mm units are utilized for representing angles and camera height, respectively. In the simulation environment, known 3D world points on planar surfaces were projected onto two-view images with the size of 1920×1200 by using similar intrinsic parameters to the real case, and Gaussian noise with zero mean and standard deviation of σ was added to the 2D projected image points. To generate the synthetic two-view images, we utilized the vehicle motion when the vehicle moves 1000 mm in forward direction as presented in Figure 4. From the 2D noisy correspondences, reconstruction methods were utilized to recover their 3D points in the camera coordinate, and the proposed calibration method was applied to estimate extrinsic parameters. Each experiment was conducted 100 times, and averaged absolute errors were measured for both reconstructed 3D points and estimated extrinsic parameters.

To evaluate reconstruction accuracy, root-mean-square errors between 3D estimates and their true positions were measured in three dimensional space. Table 1 presents reconstruction errors with respect to various amounts of noise on 2D image projections; the standard deviation σ of the Gaussian pixel-noise was varied from 0.1 to 3.0. We present two cases of simulation results: In one case, virtual planar boards are located at the longitudinal distance of around 8 m from the camera at the first view, and in the other case, those are located around 10 m. With an identical amount of pixel-noise, reconstruction error increases as the distance to the planar boards increases. Although reconstruction error increases as the amount of pixel-noise increases, the proposed reconstruction method consistently shows higher accuracies compared to the other methods. Furthermore, Table 1 demonstrates that joint optimization of one SDP for the four planes is more advantageous than separate optimization of two independent SDPs for upper two planes and lower two planes. It is because normal vectors of planes in upper and lower groups are not associated with a coplanarity constraint in the case of K=2. This result implies that joint reconstruction of entire planar surfaces is effective to reduce the reconstruction error.

Figure 4 shows the reconstruction and calibration errors in the case that distances to targets were around 8 m and the standard deviation of pixel noise was σ=0.5. By reducing the reconstruction error, calibration error was significantly decreased especially for pitch angle and camera height. In the results of the proposed method, the reconstruction and calibration errors of K=4 case were lower than those of K=2 case. It implies that increasing the number of planes was beneficial to reduce the amount of errors. However, it was not practical to setup more than four planes in real experiments, because 2D image features projected from planes which were located far from the vehicle caused a large amount of pixel noise. Therefore, we utilized four planar surfaces for extrinsic calibration in real experiments.

In the proposed reconstruction method, ego-motion was assumed as a planar motion to formulate a coplanarity constraint with plane normal vectors. To analyze the effect of vehicle motion noise to the performance of the proposed method, we conducted simulation experiments with and without vehicle motion noise. The motion noise was modeled as a Gaussian distribution, and we utilized the standard deviation of ego-motions measured in real driving scenarios to generate Gaussian motion noise in the simulation environment. Table 2 presents calibration accuracies with and without vehicle motion noise under various amounts of pixel noise. Although calibration errors were increased by the ego-motion noise, experimental results show that the proposed method was robust compared to previous methods even under the motion noise.

### 4.2. Real Calibration Environment

**Figure 5:**
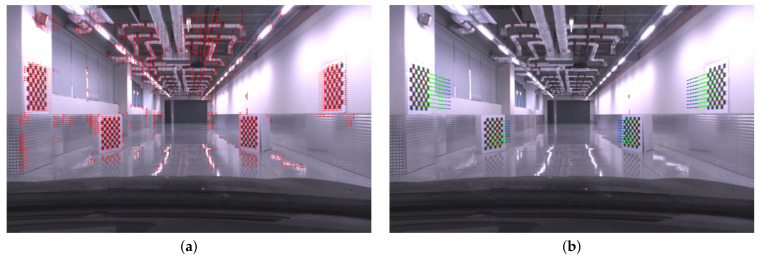
Real calibration environment: (**a**) optical flow of background features for estimating ego-motion of the vehicle. (**b**) Optical flow of chessboard features for 3d reconstruction.

This section presents experimental results in a real calibration environment to demonstrate the effectiveness of the proposed method. In our garage, chessboards were installed so that they are perpendicular to the ground as shown in Figure 5. While a vehicle moved smoothly, images were collected with the size of 1920 × 1200 by utilizing an in-vehicle frontal camera, FLIR Point Grey Grasshopper 3. Intrinsic parameters of the camera were computed in advance by using the method presented in [30]. Background features were extracted and tracked by grid-based feature detection and Lucas–Kanade method [31], and the essential matrix was computed by the five-point method [32] with RANSAC [33] to estimate camera motion. The correspondences of chessboard features were independently detected, and yaw angle of the camera with respect to moving direction was calculated based on focus of expansion, which was computed from the chessboard features. The multiple planar reconstruction method was applied to recover 3D structures of chessboard features, and finally, camera extrinsic parameters were estimated by using the proposed calibration method. This calibration process was performed multiple times while a vehicle was passing the chessboards, and these estimates were averaged to compute a final calibration parameters. In our experiment, the vehicle moved about 5 km/h to obtain enough number of image pairs, and the averaged values of 10 estimates were utilized as final extrinsic parameters. The number of calibration trials could be affected by vehicle speed, field of view of the camera, and distances between chessboards.

To evaluate the accuracy of the proposed method, we collected reference values of extrinsic parameters from an identical experiment environment. In the procedure for generating reference parameters, we manually measured 3D locations of multiple feature points with respect to the world coordinate using a laser range finder, and corresponding 2D projections in the image domain were labelled. 2D-3D correspondences were used to solve a Perspective-n-Point (PnP) problem to compute extrinsic parameters. All the procedures took around 30 min, and it was repeated eight times to obtain averaged extrinsic parameters; the reference values for camera height, pitch angle, roll angle are 1195.48 mm, 0.2413 degree, 0.3663 degree, respectively. In the real experiment, we measured absolute errors between the reference parameters and estimated extrinsic parameters.

**Figure 6:**
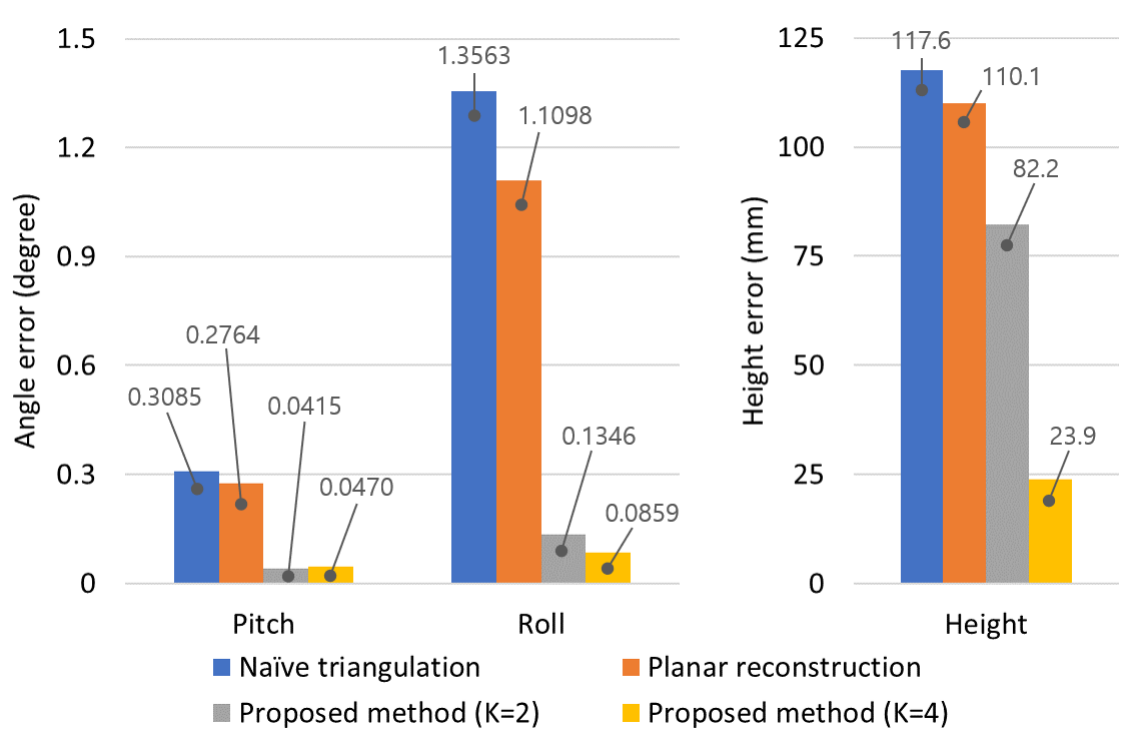
Calibration errors in real calibration scenario.

To demonstrate the effectiveness of the proposed method, we conducted experiments with four different reconstruction methods: Naïve triangulation [28] and optimal planar reconstruction method [13], and the proposed constrained multiple planar reconstructions (K=2 and K=4). Calibration errors in the real calibration scenario are presented in Figure 6, and the proposed reconstruction method gives much lower calibration errors compared to the conventional methods. Similar to experimental results in synthetic simulation, calibration accuracy was improved by utilizing a greater number of planar surfaces in a SDP. Compared to the previous planar reconstruction method [13], height error of the proposed method (K=4) was reduced from 110.1 mm to 23.9 mm, and pitch angle and roll angle errors were reduced from 0.2764 degree to 0.0470 degree and from 1.1098 degree to 0.0859 degree, respectively; about 78% and 87% of height and angle errors were reduced by using the coplanarity constraint. Because angle errors less than 0.1 degree and height error less than 30 mm were not significant to perform high-level functions such as distance estimation and lane departure warning, the proposed method was able to be utilized in intelligent vehicle industries for computing extrinsic parameters between the camera coordinate and the world coordinate.

## 5. Conclusions

In this paper, we propose a method for automatic camera calibration of intelligent vehicles. The proposed method is based on 3D reconstruction of a man-made environment, and the key contribution of this paper is novel multiple planar reconstruction method to reduce errors in camera extrinsic parameters. We first formulate a QCQP with the coplanarity constraints between plane normal vectors and camera motion vector. The QCQP is reformulated into an SDP, and the optimal solution is obtained using rank-1 relaxation. From the optimal solution of the relaxed SDP, normal vectors are computed for 3D reconstruction of planar surfaces.

We also propose a method to compute camera extrinsic parameters by utilizing planar surfaces which are perpendicular to the ground. This man-made environment is quite reasonable for end-of-line calibration and indoor camera calibration. Main benefit of the proposed method is that it does not require 3D measurements of image features, and thus, extrinsic calibration can be conducted automatically at the start of every driving. In both synthetic simulation and real calibration environment, the proposed reconstruction method significantly outperformed the previous 3D reconstruction methods, and thus errors in extrinsic parameters were dramatically reduced.

## Figures and Tables

**Table 1:** Reconstruction errors (mm) with respect to various amounts of pixel noise.

Method	Distance	σ								
		0.1	0.2	0.3	0.5	0.7	1.0	1.5	2.0	3.0
Naïve triangulation	8 m	15.7	30.8	46.6	78.0	110.4	156.0	236.6	318.2	474.8
Optimal triangulation		15.7	30.8	46.7	78.0	110.4	156.0	236.5	317.9	473.8
Planar reconstruction		3.7	7.3	11.4	17.9	26.5	37.7	58.0	77.3	119.5
Propoased method (K = 2)		3.2	6.2	9.8	15.2	22.4	32.4	51.2	70.6	110.5
Propoased method (K = 4)		3.0	5.8	9.1	14.1	20.5	30.1	48.1	67.9	106.3
Naïve triangulation	10 m	30.1	60.7	90.3	152.4	210.6	301.0	455.4	610.2	938.5
Optimal triangulation		30.1	60.7	90.3	152.4	210.5	300.9	455.1	609.1	935.4
Planar reconstruction		6.9	13.9	21.0	34.7	49.3	70.2	108.8	151.9	252.2
Propoased method (K = 2)		6.0	11.6	18.3	30.0	43.0	62.3	99.4	140.8	240.1
Propoased method (K = 4)		5.6	10.9	17.2	28.0	40.1	60.6	95.8	138.0	237.4

**Table 2:** Calibration accuracy in simulation environment with respect to various amounts of pixel noise.

Method	σ=0.3			σ=0.5			σ=0.7			σ=1.0		
	Pitch	Roll	Height	Pitch	Roll	Height	Pitch	Roll	Height	Pitch	Roll	Height
	Without camera-motion noise											
Naïve triangulation	0.9829	0.2859	141.3	1.3705	0.4759	197.3	2.4338	0.9065	349.8	3.3727	2.2364	483.3
Planar reconstruction	0.6383	0.2017	91.7	1.0972	0.3225	158.0	1.6317	0.3994	234.8	2.2217	0.7261	319.5
Propoased method (K = 2)	0.0372	0.2048	5.3	0.0751	0.3295	10.9	0.1647	0.4021	23.6	0.3394	0.6990	48.8
Propoased method (K = 4)	0.0312	0.1587	4.5	0.0557	0.2549	8.0	0.0683	0.2890	9.8	0.1330	0.5153	19.1
	With camera-motion noise											
Naïve triangulation	3.0058	0.3214	432.7	3.4568	0.5304	498.9	4.5159	0.9182	646.8	4.9208	1.7227	704.0
Planar reconstruction	2.8668	0.2067	412.8	2.9781	0.3346	429.9	3.8155	0.4555	547.0	4.0049	0.6995	574.8
Propoased method (K = 2)	0.0703	0.2152	10.2	0.1290	0.3486	18.3	0.2741	0.4797	39.4	0.4505	0.7181	65.2
Propoased method (K = 4)	0.0587	0.1501	9.2	0.1076	0.2538	15.1	0.2200	0.3271	31.7	0.3097	0.5023	44.2

## Data Availability

Not applicable.
